# An Alternative to the Human Hemoglobin Test in the Investigation of Bloodstains Treated with Active Oxygen: The Human Glycophorin A Test

**DOI:** 10.1100/tsw.2011.89

**Published:** 2011-04-19

**Authors:** Ana Castelló, Francesc Francés, Fernando Verdú

**Affiliations:** Department of Legal and Forensic Medicine, Faculty of Medicine, University of Valencia, Spain

**Keywords:** forensic sciences, bloodstains investigation, human hemoglobin test, human glycophorin A test

## Abstract

In criminal investigations, there are three stages involved when studying bloodstains: search and orientation, confirmation, and individualization. Confirmatory tests have two aims: to show that the stain contains a human biological fluid and to confirm the type of biological fluid. The need to determine the nature of the evidence is reflected in the latest bibliography, where the possibility of employing mRNA and miRNA markers for this purpose is proposed. While these new proposals are being investigated, the kits for determining human hemoglobin currently provide a simple solution for resolving this issue. With these kits, the possibility of obtaining false positives and false negatives is well known. However, recently, a new problem has been detected. This involves the interference caused by new cleaning products that contain sodium percarbonate (or active oxygen) when determining human hemoglobin. With the aim to resolve this problem, this work studied the ability of the human glycophorin A test to determine human blood in samples that have been treated with active oxygen. Our results show that the human glycophorin A test has a greater resistance to the destructive effect of the new detergents containing active oxygen; consequently, it provides an alternative to be taken into consideration in the confirmatory diagnoses of bloodstains.

### INTRODUCTION

In criminal investigations, there are three successive stages involved when studying bloodstains: search and orientation, confirmation, and individualization[1]. The first stage involves tests, called presumptive tests, which are responsible for locating latent remains and providing some preliminary information on the possible blood content of the sample. The second stage consists of establishing the origin of the stain. The question to be answered here is: Is this really a human stain? Confirmatory tests have to be undertaken for two reasons:

1. To show that the stain contains human biological fluid. By undertaking this test, one can avoid performing a lengthy and expensive genetic analysis on stains that may appear to be blood, but are not.
2. To confirm the type of biological fluid that has been found. Clearly, biological samples are destined for genetic analysis, but to discover the type of fluid under consideration, it is also essential to reconstruct and understand the events.

The need to determine the nature of the evidence is reflected in the latest bibliography that includes interesting studies on the possibility of employing mRNA[2,3] and miRNA markers[4] for this purpose. Other procedures, although still being researched, are evaluating the use of RAMAN spectroscopy as a simple and nondestructive alternative to discovering the origin of the sample[5,6]

While these new proposals are being investigated, the kits for determining human hemoglobin currently provide a simple and rapid solution for resolving this issue. In addition, they only require a small amount of sample that can be reused in the later genetic analysis[7]. In general, they are less sensitive than the presumptive test, although more specific. The possibility of obtaining false positives (by cross-reaction) and false negatives (caused by low concentration of blood in the sample or, on the contrary, by an excessively high concentration that generates the known “hook effect”) is well known[7,8,9].

However, recently, a new cause of error has been added. This involves the interference caused by new cleaning products that contain sodium percarbonate (also known as active oxygen) when determining human hemoglobin. Stains that contain an appreciable amount of blood have clearly been shown to provide a negative result to the test after being washed with this type of product, even though they remain visible[10]. However, in 2010, an article showed that the washing of bloodstains with active oxygen does not impede DNA analysis[11].

It is necessary, therefore, to look for an alternative to the hemoglobin kits in order to resolve this problem. Since 2008, a kit has been available for determining human glycophorin A[1] that, according to the studies published so far, is effective at diagnosing human blood[12,13,14].

This work aims to evaluate the ability of that protein to determine human blood in samples that have been submitted to the effect of active oxygen. For this purpose, the following experimental procedure was designed.

### MATERIALS AND METHODS

#### Materials

- Supports for bloodstains (white cotton fabric)
- Human blood (newly extracted blood with no preservatives)

#### Reagents

- Luminol (3-aminophthalhydrazide) (Merck)
- Sodium perborate (Panreac)
- Sodium carbonate (Panreac)
- Distilled water
- Bluestar® Forensic

[1]Perhaps it could be useful to remember that human glycophorin A is a sialoglycoprotein of the human erythrocyte membrane, involved in the cells’ interactions with others cells and external agents.

- Hexagon OBTI® test
- Rapid Stain Identification™-Blood (RSID™-Blood)
- Sodium percarbonate (Panreac)

### Methods

Three different tests were undertaken:

- Test 1: This test evaluates the effect of detergents containing active oxygen on determining human hemoglobin and glycophorin A (using the Hexagon OBTI test and RSID-Blood, respectively).
- Test 2: This test involves the same procedures used in Test 1, but using blood stains of different dilution (decreasing).
- Test 3: This test involves the same procedures as above, but, in this case, the concentration of active oxygen used to wash the stains was varied.

#### *Test 1*

##### Sample Preparation

Bloodstains were prepared using newly extracted blood via venipuncture, obtained from volunteers. No preservatives were added.

Stains were made on pieces of white cotton fabric of approximately 5 × 5 cm. Each stain was made with three drops of blood. Having made the stains, they were left to dry over 24 h at room temperature, without any protection whatsoever.

Once dry, the stains were divided into four groups – A, B, C, and D – that were submitted to the following treatments:

- **A Samples** — Washing with a detergent containing active oxygen[2] following the manufacturer's instructions for hand washing. The fabric was left in hot water (at 40ºC), with the cleaning product for 2 h. It was then rinsed in running water and left to dry for a day. Figs. 1 and 2 show one of the bloodstains, before and after washing.
- **B Samples** — Washing with active oxygen and in cold water for 2 h. It was then rinsed in running water and left to dry. These samples were used to determine possible differences in the results obtained from those using the product in hot water.
- **C Samples** — Washing in hot water (at 40ºC) for 2 h, without using detergent, in order to determine the possible influence of water temperature on test results. As with samples A and B, they were left to dry for 1 day.
- **D Samples** — These were not washed. They were used as control samples.

Each stain was then divided, using sterile scalpels, into four equal portions. One was used for the luminol test, another for the Bluestar Forensic test, and the other two were used for undertaking the human hemoglobin and glycophorin A tests.

[2]The product used was Neutrex™. The composition of the product, as it appears on the label, is as follows: nonionic and anionic tensoactive agents, polycarboxilates, zeolites (concentration <5%), and sodium percarbonate (in a proportion >30%). The pH of the product dissolved in water is 10.

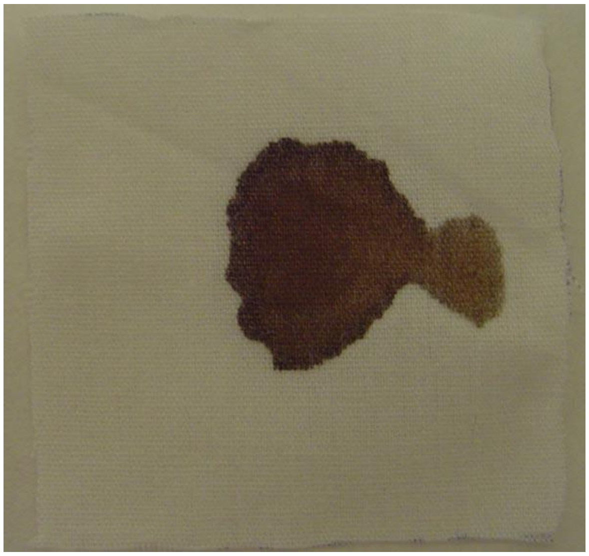

**FIGURE 1.** Bloodstain before washing.

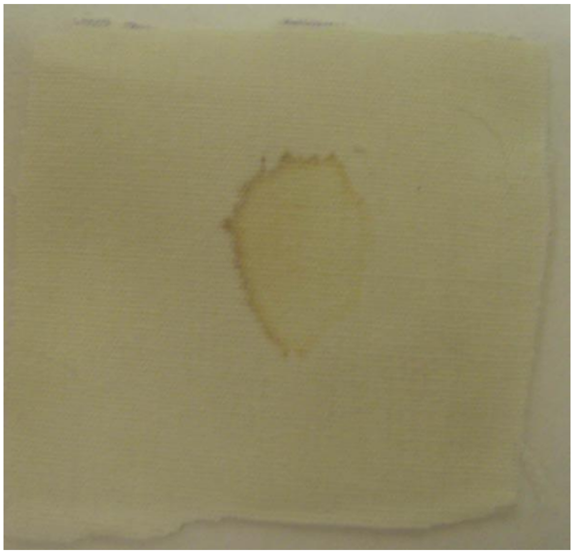

**FIGURE 2.** Bloodstain after washing.

The procedures for carrying out the tests were as follows:

- **Luminol test** — Luminol solution was prepared in accordance with the Grodsky formula[15]. This was performed in a darkroom. The reagent was then sprayed onto the stain.
- **Bluestar Forensic** — The reagent was prepared following the manufacturer's instructions[3]. Like luminol, Bluestar Forensic requires working in a darkroom where the reagent is applied using a spray gun on the stain.

[3]http://www.bluestar-forensic.com/ (accessed on February 2011).

The two tests (luminol and Bluestar Forensic) were undertaken on stain groups A, B, C, and D (as positive control) and also on support pieces (white cotton material) without stains (negative control). Tests were considered positive when the luminescence was observed a few seconds after applying the reagent.

- **Human hemoglobin test** — The Hexagon OBTI test kit, widely used in forensic laboratories, was used. The test was performed following the manufacturer's instructions[4]. The result was regarded as positive when, as stated in the manufacturer's instructions, the blue test line (indicating the positive result) formed in the first 5 min following the beginning of the test. Negative results were confirmed at 10 min.
- **Human glycophorin A test** — The RSID-Blood test was used for human glycophorin A determination. Samples were tested according to the RSID-Blood protocols[5]. Only the proportion between the amount of blood and the blood running buffer was changed, in such a way that instead of mixing 20 µl of sample with 80 of buffer, the proportion was 50%. This change was introduced with the aim of reducing the dilution of the sample. The result was regarded as positive when, as stated in the manufacturer's instructions, the red test line (indicating the positive result) formed in the first 10 min following the beginning of the test. Negative results were confirmed at 20 min.

In total, 50 stains of each type (A, B, C, and D) were processed.

### Test 2

Blood solutions (concentration 1:50, 1:100, 1:200, 1:400, 1:600, 1:800, 1:1000, expressed in milliliters of blood per total milliliters volume) were prepared using newly extracted blood, obtained from volunteers via venipuncture. No preservatives were added.

Stains were prepared from each of the solutions, following the same procedure as described in test 1. Samples A, B, C, and D were submitted to luminol, Bluestar Forensic, human hemoglobin, and human glycophorin A tests, as described in test 1.

In total, 50 stains of each type (A, B, C, and D) were processed.

### Test 3

In this case, the stains were obtained following the procedure described in test 1 from undiluted blood.

To evaluate the effect of different concentrations of the detergent, different solutions of sodium percarbonate were prepared, as this chemical compound is the main active agent of the product. The concentration of the sodium percarbonate solution was calculated from the information stated on the detergent label (Neutrex™). This involved:

- Amount of product recommended for hand washing: 80 g of detergent for 4 l of water.
- Sodium percarbonate concentration: 30%.

To wash the stains, an initial solution was prepared by diluting 0.3 g of sodium percarbonate in 50 ml of distilled water (solution concentration, $2 \times 10^{-2}$ M). By doing so, a reagent concentration equivalent to that of the original product was obtained.

[4]http://www.bluestar-forensic.com/gb/documentation_hexagon.php (accessed on February 2011)
[5]http://www.ifi-test.com/rsid_blood.php (accessed on February 2011)

From that base solution (hereafter referred to as 1/1), other diluted solutions were prepared to concentrations of 1/50, 1/100, 1/200, 1/400. The stains were washed following the procedure described in test 1, but substituting the detergent for percarbonate solutions.

Samples A, B, C, and D were submitted to the same tests (luminol, Bluestar Forensic, human hemoglobin, and human glycophorin A test) as described in test 1.

Again 50 stains of each type were processed (A, B, C, and D).

### RESULTS AND DISCUSSION

The results of each test are summarized in Tables 1–5 (Appendix). Here a positive test is represented as "++" when the reaction is intense and as "+" for a low-intensity reaction; a negative test is represented as "-".

On the data obtained in each test, the following should be borne in mind.

#### Test 1

From the results for the A, B, C, and D samples, which are summarized in Table 1, it can be deduced that:

- All the A samples processed (washed with sodium percarbonate, 40ºC) have provided negative results for the luminol, Bluestar Forensic, and human hemoglobin tests. Only the human glycophorin A test gave positive results on all the stains from this group. Consequently, washing with active oxygen interferes in the orientation tests, as well as in determining human hemoglobin by means of the Hexagon OBTI test.
- All the B samples processed (washed with sodium percarbonate, cold water) have provided positive results for all the tests undertaken with the exception of the human hemoglobin test. To correctly interpret these results, it is important to point out that the product with active oxygen does not dissolve easily in cold water. Only by agitating the solution can a partial dissolution be achieved. Consequently, it is to be expected that it is not as effective as when used following the manufacturer's instructions (water at approximately 40ºC).
- For the C samples (water 40ºC), all results are positive, so it may be deduced that washing in hot water does not reduce the efficacy of the tests undertaken. These data strengthen the hypothesis that active oxygen is responsible for the negative results obtained in the A samples.
- The D samples, used as controls, provided positive results for all tests.

#### Test 2

The data for A and B samples are summarized in Tables 2 and 3. Given that the results for C and D samples are all positive, it was not considered necessary to include a table for them. From the data obtained, it should be noted that:

- All the A samples processed (washed with sodium percarbonate, 40ºC) provided negative results to the luminol, Bluestar Forensic, and human hemoglobin tests. For the human glycophorin A test, the results were positive (to different degrees of intensity) up to a dilution of 1/1000, when the test provides a negative result. Consequently the human glycophorin A test resists the treatment of washing low-concentration bloodstains with active oxygen.
- All B samples (washed with sodium percarbonate, cold water) provided positive results for the human glycophorin A test. The tests with luminol and Bluestar Forensic provided positive results in less diluted stains (1/1, 1/50, and 1/100). However from 1/200 upwards, results are negative after washing. The human hemoglobin test was negative in all B samples. Again, it is important to point out that the product with active oxygen does not dissolve easily in cold water. Only by agitating the solution can a partial dissolution be achieved. Consequently, it is to be expected that it is not as effective as when used following the manufacturer's instructions (water at approximately 40ºC).

- For C samples (water 40ºC), all results were positive, so it can be deduced that washing in hot water does not reduce the efficacy of the tests employed, not even when blood concentration is low.
- D samples, used as controls, provided positive results for all tests.

#### Test 3

The results for A and B samples are summarized in Tables 4 and 5. Given that the results for C and D samples are all positive, it was not considered necessary to include a table for them. From the data obtained, it can be deduced that:

- For the human glycophorin A test, the results were positive for all concentrations of active oxygen tested. On reducing the concentration of active oxygen to 1/100, positive results were obtained for luminol and Bluestar Forensic. The human hemoglobin test was only positive for a concentration of contaminant equal to or less than 1/400.
- All the B samples processed (washed with sodium percarbonate, cold water) provided positive results for the luminol, Bluestar Forensic, and human glycophorin A tests. For human hemoglobin, positive results were obtained on reducing the concentration of contaminant to half the original quantity. These results concur, as is to be expected, with those obtained in test 1 for this group of samples.
- For C (water 40ºC) and D samples (control), all results were positive.

### CONCLUSION

From the results obtained, it may be concluded that human glycophorin A is an effective marker for identifying human blood. In comparison with hemoglobin, glycophorin A shows greater resistance to the destructive effect of the new detergents containing active oxygen, so providing an alternative should be taken into consideration in the certain diagnoses of bloodstains.

Perhaps the different function of both proteins (hemoglobin is able to break down hydrogen peroxide, but that is not a function of glycophorin A) could explain the different resistance to the "active oxygen effect". However, to be able to confirm this hypothesis, more studies must be made.

### ACKNOWLEDGMENTS

We give our thanks to Antonio Cantu (Chief Research Scientist, retired, of the Department of Homeland Security, U.S. Secret Service) for his help correcting and improving the translation of the text.

## References

1. Virkler, K. and Lednev, I.K. (2009) Analysis of body fluids for forensic purposes: from laboratory testing to non-destructive rapid confirmatory identification at a crime scene. *Forensic Sci. Int.* **188,** 1–17.

2. Zubakov, D., Kokshoorn, M., Kloosterman, A., and Kayser, M. (2009) New markers for old stains: stable mRNA markers for blood and saliva identification from up to 16-year-old stains. *Int. J. Legal Med.* **123,** 71–74.
3. Vennemann, M. and Koppelkamm, A. (2010) mRNA profiling in forensic genetics I: possibilities and limitations. *Forensic Sci. Int.* **203,** 71–75.
4. Courts, C. and Madea, B. (2010) Micro-RNA–A potential for forensic science? *Forensic Sci. Int.* **203**, 106–111.
5. Virkler, K. and Lednev, I.K. (2009) Blood species identification for forensic purposes using Raman spectroscopy combined with advanced statistical analysis. *Anal. Chem*. **81,** 7773–7777.
6. Virkler, K. and Lednev, I.K. (2010) Raman spectroscopic signature of blood and its potential application to forensic body fluid identification. *Anal. Bioanal. Chem*. **396,** 525–534.
7. Johnson, E., Ames, C.E., Dagnall, K.E., Foster, J., and Daniel, B.E. (2008) Comparison of presumptive blood test kits including Hexagon OBTI. *J. Forensic Sci.* **53(3),** 687–689.
8. Hochmeister, M.N., Budowle, B., Sparkes, R., Rudin, O., Gehrig, C., Thali, M., et al. (1999) Validation studies of an immunochromatographic 1-step test for the forensic identification of human blood. *J. Forensic Sci.* **44(3),** 597–602.
9. Hermon, D., Shpitzen, M., Oz, C., Glattstein, B., Azoury, M., and Gafny, R. (2003) The use of the Hexagon OBTI test for detection of human blood at crime scenes and on items of evidence. Part I: validation studies and implementation. *J. Forensic Identification* **53(5),** 566–575.
10. Castelló, A., Francès, F., Corella, D., and Verdú, F. (2009) Active oxygen doctors the evidence. *Naturwissenschaften* **96,** 303–307.
11. Castelló, A., Francés, F., and Verdú, F. (2010) DNA evidence uncompromised by active oxygen. *TheScientificWorldJOURNAL* **10,** 387–392.
12. Thorogate, R., Moreira, J.C.S., Jickells, S., Miele, M.M.P., and Daniel, B. (2008) A novel fluorescence-based method in forensic science for the detection of blood in situ. *Forensic Sci. Int. Genet.* **2,** 363–371.
13. Schweers, B.A., Old, J., Boonlayangoor, P.W., and Reich, K.A. (2008) Developmental validation of a novel lateral flow strip test for rapid identification of human blood (Rapid Stain IdentificationTM-Blood). *Forensic Sci. Int. Genet.* **2,** 243–247.
14. Turrina, S., Filippini, G., Atzei, R., Zaglia, E., and De Leo, D. (2008) Validation studies of rapid stain identification-blood (RSID-blood) kit in forensic caseworks. *Forensic Sci. Int. Genet. Suppl. Ser.* **1,** 74–75.
15. Grodsky, M., Wright, K., and Kirk, P.L. (1951) Simplified preliminary blood testing. An improved technique and comparative study of methods. *J. Crim. Law Criminol. Police Sci.* **42,** 95–104.

## APPENDIX

**TABLE 1**
**Results of Luminol, Bluestar Forensic, Human Hemoglobin, and Human Glycophorin A Tests on Bloodstains Washed with Detergents Containing Active Oxygen**

| Test | Samples | | | |
|---|---|---|---|---|
| | A | B | C | D |
| Luminol | - | ++ | ++ | ++ |
| Bluestar Forensic | - | ++ | ++ | ++ |
| Human hemoglobin test (Hexagon OBTI test) | - | - | ++ | ++ |
| Human glycophorin A test (RSID-Blood) | ++ | ++ | ++ | ++ |

A = Washed with sodium percarbonate, 40ºC; B = washed with sodium percarbonate, cold water; C = water 40ºC; D = control. ++ = Positive, intense reaction; + = positive, low-intensity reaction; - = negative.

**TABLE 2**
**Results of the Luminol, Bluestar Forensic, Human Hemoglobin, and Human Glycophorin A Tests on Bloodstains Prepared with Different Dilutions of Blood and Washed with Sodium Percarbonate at 40ºC (A Samples)**

| Test | Blood Solution (ml blood/ml solution) | | | | | | | |
|---|---|---|---|---|---|---|---|---|
| | 1/1 | 1/50 | 1/100 | 1/200 | 1/400 | 1/600 | 1/800 | 1/1000 |
| Luminol | - | - | - | - | - | - | - | - |
| Bluestar Forensic | - | - | - | - | - | - | - | - |
| Human hemoglobin test (Hexagon OBTI test) | - | - | - | - | - | - | - | - |
| Human glycophorin A test (RSID-Blood) | ++ | ++ | ++ | + | + | + | + | - |

++ = Positive, intense reaction; + = positive, low-intensity reaction; - = negative.

**TABLE 3**
**Results of the Luminol, Bluestar Forensic, Human Hemoglobin, and Human Glycophorin A Tests on Bloodstains Prepared with Different Dilutions of Blood and Washed with Sodium Percarbonate in Cold Water (B Samples)**

| Test | Blood Solution (ml blood/ml solution) | | | | | | | |
|---|---|---|---|---|---|---|---|---|
| | 1/1 | 1/50 | 1/100 | 1/200 | 1/400 | 1/600 | 1/800 | 1/1000 |
| Luminol | ++ | + | + | - | - | - | - | - |
| Bluestar Forensic | ++ | ++ | + | - | - | - | - | - |
| Human hemoglobin test (Hexagon OBTI test) | - | - | - | - | - | - | - | - |
| Human glycophorin A test (RSID-Blood) | ++ | ++ | ++ | ++ | ++ | + | + | + |

++ = Positive, intense reaction; + = positive, low-intensity reaction; - = negative.

**TABLE 4**
**Results of the Luminol, Bluestar Forensic, Human Hemoglobin, and Human Glycophorin A Tests on Bloodstains Washed in Water at 40ºC, with Sodium Percarbonate to Different Concentrations (A Samples)**

| Test | Sodium Percarbonate Solutions | | | | |
|---|---|---|---|---|---|
| | 1/1* | 1/50 | 1/100 | 1/200 | 1/400 |
| Luminol | - | ++ | ++ | ++ | ++ |
| Bluestar Forensic | - | ++ | ++ | ++ | ++ |
| Human hemoglobin test (Hexagon OBTI test) | - | - | - | - | + |
| Human glycophorin A test (RSID-Blood) | ++ | ++ | ++ | ++ | ++ |

++ = Positive, intense reaction; + = positive, low-intensity reaction; - = negative.

* 1/1 corresponds to the concentration recommended by the manufacturer, 0.3 g of sodium percarbonate in 50 ml of distilled water (solution concentration, $2 \times 10^{-2}$ M). All the others were obtained from that dilution base.

**TABLE 5**
**Results of the Luminol, Bluestar Forensic, Human Hemoglobin, and Human Glycophorin A Tests on Bloodstains Washed in Cold Water with Sodium Percarbonate to Different Concentrations (B Samples)**

| Test | Sodium Percarbonate Solutions | | | | |
|---|---|---|---|---|---|
| | 1/1* | 1/50 | 1/100 | 1/200 | 1/400 |
| Luminol | + | ++ | ++ | ++ | ++ |
| Bluestar Forensic | + | ++ | ++ | ++ | ++ |
| Human hemoglobin test (Hexagon OBTI test) | - | ++ | ++ | ++ | ++ |
| Human glycophorin A test (RSID-Blood) | ++ | ++ | ++ | ++ | ++ |

++ = Positive, intense reaction; + = positive, low-intensity reaction; - = negative.

* 1/1 corresponds to the concentration recommended by the manufacturer, 0.3 g of sodium percarbonate in 50 ml of distilled water (solution concentration, $2 \times 10^{-2}$ M). All the others were obtained from that dilution base.
